# Supplementary material for: Evolution of cation binding in the active sites of P-loop nucleoside triphosphatases in relation to the basic catalytic mechanism
Source: eLife. 2018 Dec 11;7:e37373. doi: 10.7554/eLife.37373 (PMC6310460; doi:10.7554/eLife.37373)
Supplement: Supplementary file 1. — (A) Monovalent cation requirements of P-loop GTPases and ATPases. (B) Properties of monovalent cations and their interactions with the Mg2+-ATP complex. (C) Molecular dynamics simulations performed in this work. (D) Values of dihedral angles of the phosphate chains of Mg-ATP in the presence of K+ ions. (E) Lifetimes of the βγ-conformation of Mg-ATP complex during MD simulations. (F) Characteristics of the triphosphate chain for different interactions between the Mg2+ ion and ATP. (G) Comparison of the PA-PG distance measurements of the βγ-coordinated Mg-ATP complexes. (H) Comparison of the PA-PG distance measurements of the αβγ-coordinated Mg-ATP complexes. (I) Comparison of the PA-PG distance measurements for the αβγ-coordinated and ‘curled’ βγ-coordinated Mg-ATP complexes in different systems. (J) Comparison of the PB-O3B-PG angle measurements for the βγ-coordinated Mg-ATP complexes. (K) Comparison of the PB-O3B-PG angle measurements for the αβγ-coordinated Mg-ATP complexes. (L) Comparison of the PA-PG distance measurements for the αβγ-coordinated and ‘curled’ βγ-coordinated Mg-ATP complexes. [file elife-37373-supp1.docx]

**Supplementary Materials**

**to Shalaeva *et al*. “Evolution of cation binding in the active sites of P-loop nucleoside triphosphatases”**

**Supplementary file 1A. Monovalent cation requirements of P-loop GTPases and ATPases**

| **TRAFAC class** | | | |
| --- | --- | --- | --- |
| **Protein name** | **UniProt ID** | **Cation dependence** | **Reference** |
| Dynamin-1 | DYN1_HUMAN | K^+^>Na^+^ | (1) |
| Dynamin-related protein 1A | DRP1A_ARATH | K^+^, Na^+^ | (2) |
| GTPase Nug1 | G0SEW3_CHATD | K^+^>Na^+^ | (3) |
| Ribosome biogenesis GTPase A | RBGA_BACSU | K^+^, no Na^+^ | (4) |
| Ribosome biogenesis GTPase RsgA (YjeQ) | RSGA_ECOLI | K^+^ | (5) |
| Elongation Factor Tu, *E. coli* | EFTU1_ECOLI | K^+^>Na^+^ | (6) |
| Elongation Factor Tu, *Haloarcula marismortui* | EF1A_HALMA | K^+^>Na^+^ | (7) |
| Eukaryotic translation initiation factor 5B | IF2P_CHATD | Na^+^, K^+^ | (8) |
| Initiation factor IF-2 | IF2_ECOLI | K^+^ | (9) |
| tRNA modification GTPase MnmE | MNME_ECOLI | K^+^, no Na^+^ | (10) |
| Ferrous iron transporter B | Q5M586_STRT2 | K^+^, no Na^+^ | (11) |
| Ribosome-binding ATPase YchF | YCHF_ECOLI | K^+^, no Na^+^ | (12) |
| GTPase HflX* | HFLX_BACSU | K^+^ | (13) |
| GTPase Era | ERA_BACSU | K^+^, no Na^+^ | (13) |
| GTP-binding protein EngA** *B. subtilis* | DER_BACSU | K^+^, no Na^+^ | (13, 14) |
| GTP-binding protein EngA** *T. maritima* | DER_THEMA | K^+^, no Na^+^ | (15) |
| NO-associated protein 1 | NOA1_ARATH | K^+^ | (16) |
| Ribosome Assembly GTPase YqeH | YQEH_BACSU | K^+^, no Na^+^ | (17) |
| Developmentally-regulated GTP-binding protein 1 | DRG1_HUMAN | K^+^ | (18) |
| GTP-binding protein EngB | ENGB_BACSU | K^+^* | (13) |
| Human GTPBP3 | GTPB3_HUMAN | K^+^ | (19) |

| **RecA-like family** | | | |
| --- | --- | --- | --- |
| Human meiotic recombinase Dmc1 | DMC1_HUMAN | K^+^ | (20) |
| Human DNA repair protein RAD51 | RAD51_HUMAN | K^+^ | (21) |
|  |  | K^+^, no Na^+^ | (22) |
| Yeast DNA repair protein RAD51 | RAD51_YEAST | K^+^ | (23) |
| DNA repair protein RadA from *M. voltae* | RADA_METVO | K^+^ | (24) |
| DNA repair protein RadA from *M. maripaludis* | RADA_METMI | K^+^, no Na^+^ | (25) |

In the ‘Cation dependence’ column, ’K^+^’ indicates that only K^+^-dependence has been shown; ’K^+^, no Na^+^’ indicates activation by K^+^ ions and a lack of activation by Na^+^ ions; ’K^+^>Na^+^’ denotes more effective activation by K^+^ than by Na^+^ ions; ’K^+^, Na^+^’ and ’Na^+^, K^+^’ is used when both cations have similar effects, with the more effective one listed first.

* The GTPase activity was measured at the same concentrations of KCl and NaCl of 200 mM, and for some proteins (the second GTPase domain of EngA, HflX, EngB, all from *B. subtilis*), the lack of activation by cations has been reported (13). However, higher concentrations of ions may be required for these proteins in the absence of their activating partners, as has been shown for the second GTPase domain of EngA (14).

** This protein has two P-loop GTPase domains, activity measurements were reported for the whole protein.

**Supplementary file 1B. Properties of monovalent cations and their interaction with the Mg^2+^-ATP complex.**

| **Cation** | **Ionic radius (Å)*^b^*** | **Stimulation of transphospho­rylation, %, *^a^*** | **Binding to ATP in the absence of Mg^2+^ (log(K_B_), 25°C** | | | **Binding to Mg-ATP (log(K_B_)*^f^*** |
| --- | --- | --- | --- | --- | --- | --- |
| Na^+^ | 1.02 | 28 | 1.31±0.03*^c^* | 1.989±0.007*^d^* | 1.93*^e^* | **2.76** |
| K^+^ | 1.38 | 64-73* | 1.17±0.03*^c^* | 1.873±0.005*^d^* | 1.99*^e^* | **0.88** |
| NH_4_^+^ | 1.44 | 27 | N/A | | | **1.76** |

* measured for different salts: 64% with KCl and 73% with K_2_SO_4_.

*a* –data from (26); stimulation of transphosphorylation by 100 mM M^+^ in the presence of 50 μM MnCl_2_.

*b* – data from (27)

*c* – data from (28)

*d –* data from (29)

*e –* data from (30)

*f* – calculated from MD simulations

**Supplementary file 1C. Molecular dynamics simulations performed in this work**

| **No.** | **System** | **Simulation time** | **Number of repetitions** |
| --- | --- | --- | --- |
| 1 | Mg-ATP | 167 ns | 3 |
| 2 | Mg-ATP, K^+^ | 167 ns | 3 |
| 3 | Mg-ATP, Na^+^ | 167 ns | 3 |
| 4 | Mg-ATP, NH_4_^+^ | 167 ns | 3 |
| 5 | Mg-ATP | 20 ns | 25 |
| 6 | Mg-ATP, K^+^ | 20 ns | 25 |
| 7 | Mg-ATP, Na^+^ | 20 ns | 25 |
| 8 | Mg-ATP, NH_4_^+^ | 20 ns | 25 |
| 9 | Mg-GTP | 20 ns | 20 |
| 10 | Mg-GTP, K^+^ | 20 ns | 20 |
| 11 | Mg-GTP, Na^+^ | 20 ns | 20 |
| 12 | Mg-GTP, NH_4_^+^ | 20 ns | 20 |
| 13 | Mg-ATP, K^+^, w. positional restraints * | 10 ns | 2 |
| 13 | Mg-GTP-MnmE, inactive, no K-loop, 3GEI | 100 ns | 1 |
| 14 | Mg-GTP-MnmE, inactive, K-loop, no K^+^, 2GJ8_W_ | 100 ns | 1 |
| 15 | Mg-GTP-MnmE, active dimer with K^+^, 2GJ8_K_ | 100 ns | 1 |

* simulations were performed with positional restrains applied to monovalent cations bound in the AG and BG sites for analysis of dihedral angles of the phosphate chainmonovalent cations bound in the AG and BG sites, see the main text for details.

**Supplementary file 1D. Values of dihedral angles of the phosphate chains of Mg-ATP in the presence of K^+^ ions.**

| **Structure** | **Ψ^α-β^** | **Ψ^β-γ^** | **Ψ^α-γ^** |
| --- | --- | --- | --- |
| Mg-ATP-K^+^  (MD simulation) | -60±23° | -4±18° | -59±35° |
| Mg-ATP-2K^+^  (MD simulation) | +13±24° | -27±8° | +1±26° |

Dihedral angle is an angle between two planes that is defined by four atoms. Values of dihedral angles between phosphate groups were defined as follows: Ψ^α-β^ = ∠O^2A^-P^A^-P^B^-O^2B^; Ψ^β-γ^ = ∠O^1B^-P^B^-P^G^-O^1G^; and Ψ^α-γ^ = ∠O^1A^-P^A^-P^G^-O^3G^, see also Fig. 3C. During the analysis of MD simulation data, the average and standard deviation values for dihedral angles were obtained by fitting the angle distribution histograms with normal functions, using the function “fit” in MatLab software (The Mathworks, Inc.). All distributions were fitted with one-term Gaussian models, except for the Ψ^β-γ^ angle in case of the Mg-ATP with two K^+^ bound; this distribution was fitted with a two-term Gaussian, and parameters are shown for the highest peak. Distribution histograms and fitted curves are shown in Figure 3C.

* The rotation of α-phosphate is unrestricted and the corresponding dihedral angles can take any values between -180° and 180°.

**Supplementary file 1E. Lifetimes of the βγ-conformation of Mg^-^ATP complex during MD simulations.**

| **Cation** | **K^+^** | **Na^+^** | **NH_4_^+^** | **no M^+^** |
| --- | --- | --- | --- | --- |
| Average lifetime (ns) | 9.49 | 10.59 | 11.04 | 9.45 |
| Standard deviation | 6.52 | 8.28 | 7.82 | 7.85 |
| Lifetime (ns) for each MD run (total run time, 20 ns) | 7.68 | 13.18 | 0.88 | 0.91 |
|  | 16.16 | 0.15 | 19.75 | 3.43 |
|  | 16.8 | 1.18 | 19.55 | 0.61 |
|  | 7.93 | 11.8 | 8.48 | 14.73 |
|  | 4.93 | 4.03 | 2.71 | **20** |
|  | 0.28 | **20** | 2.21 | 12.38 |
|  | 6.06 | **20** | 1.58 | **20** |
|  | 2.75 | 7.01 | 12.8 | 19.23 |
|  | 11.76 | 3.86 | 10.71 | 0.26 |
|  | 6.33 | 20 | 2.65 | 0.93 |
|  | 13.43 | 2.36 | **20** | **20** |
|  | 2.65 | 20 | 16.98 | 6.2 |
|  | 8.11 | **20** | 17.66 | 0.21 |
|  | 11.21 | 20 | 9.16 | 10.58 |
|  | 4.9 | **20** | **20** | 3.03 |
|  | 8.03 | 16.41 | 1.18 | 13.38 |
|  | 0.7 | 3.15 | 1.21 | 1.03 |
|  | 14.68 | 2.25 | 1.06 | 5.31 |
|  | **20** | 10.93 | 8.36 | 0.36 |
|  | **20** | 0.18 | **20** | 9.11 |
|  | 0.38 | 6.83 | 5.63 | **20** |
|  | 4.68 | **20** | **20** | 6.2 |
|  | 8.01 | 0.66 | 20 | 8.48 |
|  | **19.75** | 0.83 | 13.38 | 20 |
|  | **20** | 20 | 20 | 20 |
|  |  |  |  |  |

For each system, 25 independent 20-ns MD simulation runs were conducted, each starting with the Mg-ATP complex in the βγ conformation. Stability of the βγ conformation was tracked by measuring the distance from the Mg^2+^ ion to the nearest oxygen atom of α-phosphate, and the time periods during which the βγ conformation was retained were compared between different systems. The one-way ANOVA analysis did not reveal any significant dependence of the stability of the βγ-coordination on the monovalent cation present. For each monovalent cation, the βγ-coordination was retained during the whole 20 ns in at least four cases (shown by bold numbers). These simulations were used to characterize the shape of the phosphate chain of ATP with βγ-coordination of the Mg^2+^ ion (Table 1 and Figure 5B).

**Supplementary file 1F.** Characteristics of the triphosphate chain for different interactions between the Mg^2+^ ion and ATP.

| System | Conformation | Pα-Pγ distance, Å | Number of frames | Pβ-O-Pγ angle, ° | Number of frames |
| --- | --- | --- | --- | --- | --- |
| No cations | βγ conformation | 5.4±0.3 | 160 | 122.4±3.5 | 640 |
| K^+^ | βγ conformation | 4.9±0.2 | 85 | 128.4±3.5 | 388 |
| Na^+^ | βγ conformation | 4.8±0.1 | 109 | 128.2±3.5 | 473 |
| NH_4_^+^ | βγ conformation | 4.9±0.2 | 64 | 128.6±3.8 | 251 |
| No cations | αβγ conformation | 4.7±0.2 | 161 | 125.2±3.3 | 267 |
| K^+^ | αβγ conformation | 4.3±0.1 | 133 | 127.9±3.6 | 198 |
| Na^+^ | αβγ conformation | 4.2±0.1 | 129 | 127.9±3.8 | 192 |
| NH_4_^+^ | αβγ conformation | 4.2±0.1 | 129 | 128.1±3.7 | 190 |
| Na^+^ | “curled” conformation | 4.6±0.2 | 131 | 124.3±3.2 | 194 |
| NH_4_^+^ | “curled” conformation | 4.6±0.2 | 125 | 124.9±3.5 | 183 |

**Supplementary file 1G.** Comparison of the P^A^-P^G^ distance measurements of the βγ-coordinated Mg-ATP complexes

| System  (number of frames) | No M^+^ ions | K^+^ | Na^+^ | NH_4_^+^ |
| --- | --- | --- | --- | --- |
| No M^+^ ions  (160) | N/A | 10^-25^ | 10^-46^ | 10^-24^ |
| K^+^  (85) | 10^-25^ | N/A | 10^-7^ | 0.16* |
| Na^+^  (109) | 10^-46^ | 10^-7^ | N/A | 0.0041 |
| NH_4_^+^  (64) | 10^-24^ | 0.16* | 0.0041 | N/A |

The null hypothesis was that the P^A^-P^G^ distances in the βγ-coordinated Mg-ATP systems with different M^+^ ions added result from normal distributions with equal mean values.

* The null hypothesis is NOT rejected, no significant difference between samples

**Supplementary file 1H.** Comparison of the P^A^-P^G^ distance measurements of the αβγ-coordinated Mg-ATP complexes

| System  (number of frames) | No M^+^ ions | K^+^ | Na^+^ | NH_4_^+^ |
| --- | --- | --- | --- | --- |
| No M^+^ ions  (161) | N/A | 10^-56^ | 10^-78^ | 10^-76^ |
| K^+^  (133) | 10^-56^ | N/A | 10^-18^ | 10^-11^ |
| Na^+^  (129) | 10^-78^ | 10^-18^ | N/A | 10^-4^ |
| NH_4_^+^  (129) | 10^-76^ | 10^-11^ | 10^-4^ | N/A |

The null hypothesis was that the P^A^-P^G^ distances in the αβγ-coordinated Mg-ATP systems with different cations added result from normal distributions with equal mean values.

**Supplementary file 1I.** Comparison of the P^A^-P^G^ distance measurements for the αβγ-coordinated and “curled” βγ-coordinated Mg-ATP complexes in different systems

| System and conformation  (number of frames) | Na^+^, αβγ | Na^+^, “curled” | NH_4_^+^, αβγ | NH_4_^+^, “curled” |
| --- | --- | --- | --- | --- |
| Na^+^, αβγ  (129) | N/A | 10^-46^ | 10^-4^ | N/A |
| Na^+^, “curled”  (121) | 10^-46^ | N/A | N/A | 0.98* |
| NH_4_^+^, αβγ  (129) | 10^-4^ | N/A | N/A | 10^-42^ |
| NH_4_^+^, “curled”  (135) | N/A | 0.98* | 10^-42^ | N/A |

The null hypothesis was that the P^A^-P^G^ distances in ATP are the same in the αβγ-coordinated and “curled” βγ-coordinated Mg-ATP complexes, respectively.

* The null hypothesis is NOT rejected, no significant difference between samples

**Supplementary file 1J.** Comparison of the P^B^-O^3B^-P^G^ angle measurements for the βγ-coordinated Mg-ATP complexes

| System  (number of frames) | No M^+^ ions | K^+^ | Na^+^ | NH_4_^+^ |
| --- | --- | --- | --- | --- |
| No M^+^ ions  (640) | N/A | 10^-118^ | 10^-127^ | 10^-94^ |
| K^+^  (388) | 10^-118^ | N/A | 0.46* | 0.49* |
| Na^+^  (473) | 10^-127^ | 0.46* | N/A | 0.17* |
| NH_4_^+^  (251) | 10^-94^ | 0.49* | 0.17* | N/A |

The null hypothesis was that the P^B^-O^3B^-P^G^ angles in the βγ-coordinated Mg-ATP complexes with different M^+^ ions added result from normal distributions with equal mean values.

* The null hypothesis was NOT rejected, no significant difference between samples

**Supplementary file 1K.** Comparison of the P^B^-O^3B^-P^G^ angle measurements for the αβγ-coordinated Mg-ATP complexes

| System  (number of frames) | No M^+^ ions | K^+^ | Na^+^ | NH_4_^+^ |
| --- | --- | --- | --- | --- |
| No M^+^ ions  (267) | N/A | 10^-16^ | 10^-15^ | 10^-17^ |
| K^+^  (198) | 10^-16^ | N/A | 0.94* | 0.72* |
| Na^+^  (192) | 10^-15^ | 0.94* | N/A | 0.68* |
| NH_4_^+^  (190) | 10^-17^ | 0.72* | 0.68* | N/A |

The null hypothesis was that the P^B^-O^3B^-P^G^ angles in the αβγ-coordinated Mg-ATP complexes with different M^+^ ions added result from normal distributions with equal mean values.

* The null hypothesis was NOT rejected, no significant difference between samples

**Supplementary file 1L.** Comparison of the P^A^-P^G^ distance measurements for the αβγ-coordinated and “curled” βγ-coordinated Mg-ATP complexes

| System  (number of frames) | Na^+^, αβγ | Na^+^, “curled” | NH_4_^+^, αβγ | NH_4_^+^, “curled” |
| --- | --- | --- | --- | --- |
| Na^+^, αβγ  (192) | N/A | 10^-22^ | 0.68* | N/A |
| Na^+^, “curled”  (194) | 10^-22^ | N/A | N/A | 0.045 |
| NH_4_^+^, αβγ  (190) | 0.68* | N/A | N/A | 10^-16^ |
| NH_4_^+^, “curled”  (183) | N/A | 0.045 | 10^-16^ | N/A |

The null hypothesis was that P^A^-P^G^ distances are similar for the αβγ-coordinated and βγ-coordinated, "curled" Mg-ATP complexes.

* The null hypothesis was NOT rejected, no significant difference between samples

**References**

1. Chappie, J. S., Acharya, S., Leonard, M., Schmid, S. L., and Dyda, F. (2010) G domain dimerization controls dynamin's assembly-stimulated GTPase activity, *Nature* *465*, 435-440.

2. Yan, L., Ma, Y., Sun, Y., Gao, J., Chen, X., Liu, J., Wang, C., Rao, Z., and Lou, Z. (2011) Structural basis for mechanochemical role of *Arabidopsis thaliana* dynamin-related protein in membrane fission, *J. Mol. Cell Biol.* *3*, 378-381.

3. Manikas, R. G., Thomson, E., Thoms, M., and Hurt, E. (2016) The K^+^-dependent GTPase Nug1 is implicated in the association of the helicase Dbp10 to the immature peptidyl transferase centre during ribosome maturation, *Nucleic Acids Res.* *44*, 1800-1812.

4. Achila, D., Gulati, M., Jain, N., and Britton, R. A. (2012) Biochemical characterization of ribosome assembly GTPase RbgA in *Bacillus subtilis*, *J. Biol. Chem.* *287*, 8417-8423.

5. Daigle, D. M., and Brown, E. D. (2004) Studies of the interaction of *Escherichia coli* YjeQ with the ribosome in vitro, *J. Bacteriol.* *186*, 1381-1387.

6. Fasano, O., De Vendittis, E., and Parmeggiani, A. (1982) Hydrolysis of GTP by elongation factor Tu can be induced by monovalent cations in the absence of other effectors, *J. Biol. Chem.* *257*, 3145-3150.

7. Ebel, C., Guinet, F., Langowski, J., Urbanke, C., Gagnon, J., and Zaccai, G. (1992) Solution studies of elongation factor Tu from the extreme halophile *Halobacterium marismortui*, *J. Mol. Biol.* *223*, 361-371.

8. Kuhle, B., and Ficner, R. (2014) A monovalent cation acts as structural and catalytic cofactor in translational GTPases, *EMBO J.* *33*, 2547-2563.

9. Dubnoff, J. S., and Maitra, U. (1972) Characterization of the ribosome-dependent guanosine triphosphatase activity of polypeptide chain initiation factor IF 2, *J. Biol. Chem.* *247*, 2876-2883.

10. Scrima, A., and Wittinghofer, A. (2006) Dimerisation-dependent GTPase reaction of MnmE: how potassium acts as GTPase-activating element, *EMBO J.* *25*, 2940-2951.

11. Ash, M. R., Guilfoyle, A., Clarke, R. J., Guss, J. M., Maher, M. J., and Jormakka, M. (2010) Potassium-activated GTPase reaction in the G protein-coupled ferrous iron transporter B, *J. Biol. Chem.* *285*, 14594-14602.

12. Tomar, S. K., Kumar, P., and Prakash, B. (2011) Deciphering the catalytic machinery in a universally conserved ribosome binding ATPase YchF, *Biochem Biophys Res Commun* *408*, 459-464.

13. Rafay, A., Majumdar, S., and Prakash, B. (2012) Exploring potassium-dependent GTP hydrolysis in TEES family GTPases, *FEBS Open Bio* *2*, 173-177.

14. Foucher, A. E., Reiser, J. B., Ebel, C., Housset, D., and Jault, J. M. (2012) Potassium acts as a GTPase-activating element on each nucleotide-binding domain of the essential *Bacillus subtilis* EngA, *PLoS One* *7*, e46795.

15. Hwang, J., and Inouye, M. (2001) An essential GTPase, Der, containing double GTP-binding domains from *Escherichia coli* and *Thermotoga maritima*, *J. Biol. Chem.* *276*, 31415-31421.

16. Moreau, M., Lee, G. I., Wang, Y., Crane, B. R., and Klessig, D. F. (2008) AtNOS/AtNOA1 is a functional *Arabidopsis thaliana* cGTPase and not a nitric-oxide synthase, *J. Biol. Chem.* *283*, 32957-32967.

17. Anand, B., Surana, P., and Prakash, B. (2010) Deciphering the catalytic machinery in 30S ribosome assembly GTPase YqeH, *PLoS One* *5*, e9944.

18. Perez-Arellano, I., Spinola-Amilibia, M., and Bravo, J. (2013) Human Drg1 is a potassium-dependent GTPase enhanced by Lerepo4, *FEBS J.* *280*, 3647-3657.

19. Villarroya, M., Prado, S., Esteve, J. M., Soriano, M. A., Aguado, C., Perez-Martinez, D., Martinez-Ferrandis, J. I., Yim, L., Victor, V. M., Cebolla, E., Montaner, A., Knecht, E., and Armengod, M. E. (2008) Characterization of human GTPBP3, a GTP-binding protein involved in mitochondrial tRNA modification, *Mol Cell Biol* *28*, 7514-7531.

20. Sehorn, M. G., Sigurdsson, S., Bussen, W., Unger, V. M., and Sung, P. (2004) Human meiotic recombinase Dmc1 promotes ATP-dependent homologous DNA strand exchange, *Nature* *429*, 433-437.

21. Liu, Y., Stasiak, A. Z., Masson, J. Y., McIlwraith, M. J., Stasiak, A., and West, S. C. (2004) Conformational changes modulate the activity of human RAD51 protein, *J. Mol. Biol.* *337*, 817-827.

22. Shim, K. S., Schmutte, C., Yoder, K., and Fishel, R. (2006) Defining the salt effect on human RAD51 activities, *DNA Repair (Amst)* *5*, 718-730.

23. Rice, K. P., Eggler, A. L., Sung, P., and Cox, M. M. (2001) DNA pairing and strand exchange by the *Escherichia coli* RecA and yeast Rad51 proteins without ATP hydrolysis: on the importance of not getting stuck, *J. Biol. Chem.* *276*, 38570-38581.

24. Amunugama, R., He, Y., Willcox, S., Forties, R. A., Shim, K. S., Bundschuh, R., Luo, Y., Griffith, J., and Fishel, R. (2012) RAD51 protein ATP cap regulates nucleoprotein filament stability, *J. Biol. Chem.* *287*, 8724-8736.

25. Li, Y., He, Y., and Luo, Y. (2009) Conservation of a conformational switch in RadA recombinase from Methanococcus maripaludis, *Acta Crystallogr D Biol Crystallogr* *65*, 602-610.

26. Lowenstein, J. M. (1960) The stimulation of transphosphorylation by alkali-metal ions, *Biochem J* *75*, 269-274.

27. Sigel, A., Sigel, H., and Sigel, R. K. O., (Eds.) (2016) *The Alkali Metal Ions: Their Role for Life*, Springer.

28. Smith, R. M., Martell, A. E., and Chen, Y. (1991) Critical-Evaluation of Stability-Constants for Nucleotide Complexes with Protons and Metal-Ions and the Accompanying Enthalpy Changes, *Pure Appl Chem* *63*, 1015-1080.

29. De Stefano, C., Milea, D., Pettignano, A., and Sammartano, S. (2006) Modeling ATP protonation and activity coefficients in NaCl_aq_ and KCl_aq_ by SIT and Pitzer equations, *Biophys. Chem.* *121*, 121-130.

30. Stellwagen, E., and Stellwagen, N. C. (2007) Quantitative analysis of cation binding to the adenosine nucleotides using the variable ionic strength method: Validation of the Debye–Hückel–Onsager theory of electrophoresis in the absence of counterion binding, *Electrophoresis* *28*, 1053-1062.
